# Nutrition Literacy and Adherence to the Mediterranean Diet in Women Aged 45–70 Years: A Cross-Sectional Analysis from the Ophelia Study in Florence

**DOI:** 10.3390/nu18081238

**Published:** 2026-04-15

**Authors:** Chiara Lorini, Diletta Buresta, Chiara Marini, Claudia Cosma, Claudia Biagi, Chiara Milani, Giulia Naldini, Gabriele Cerini, Alice Graziani, Marco Del Riccio, Patrizio Zanobini, Veronica Gallinoro, Lorenzo Baggiani, Marco Nerattini, Guglielmo Bonaccorsi

**Affiliations:** 1 Department of Health Science, University of Florence, Viale G.B. Morgagni 48, 50134 Florence, Italy; chiara.lorini@unifi.it (C.L.); chiara.marini2@edu.unifi.it (C.M.); claudia.cosma@unifi.it (C.C.); claudia.biagi@unifi.it (C.B.); chiara.milani@unifi.it (C.M.); marco.delriccio@unifi.it (M.D.R.); patrizio.zanobini@unifi.it (P.Z.); guglielmo.bonaccorsi@unifi.it (G.B.); 2 Health Literacy Laboratory, Department of Health Science, University of Florence, Viale G.B. Morgagni 48, 50134 Florence, Italy; 3 Department of District Healthcare Network, Azienda USL Toscana Centro, 50122 Florence, Italy; giulia.naldini@uslcentro.toscana.it (G.N.); lorenzo.baggiani@uslcentro.toscana.it (L.B.); 4 Medical Specialization School of Hygiene and Preventive Medicine, University of Florence, 50134 Florence, Italy; gabriele.cerini@unifi.it (G.C.); alice.graziani@unifi.it (A.G.); veronica.gallinoro@unifi.it (V.G.); 5 Health Society of Florence, 50122 Florence, Italy; marco.nerattini@sds.firenze.it

**Keywords:** menopause, Mediterranean diet, nutrition literacy, dietary behavior, cardiovascular disease prevention

## Abstract

**Background/Objectives**: Nutrition literacy (NL) is an important determinant of healthy dietary behaviors, particularly among population groups at increased cardiovascular risk. This study aimed to describe NL and adherence to the Mediterranean diet (MD), and to describe their association, among women aged 45–70 years living in socioeconomically disadvantaged neighborhoods in Florence (Italy). **Methods**: A cross-sectional study was conducted within the Joint Action on Cardiovascular Diseases and Diabetes (JACARDI). This study represents the second step of Phase 1 of the Optimising Health Literacy and Access (Ophelia) process. Women were recruited in a primary health care setting using a convenience sample. NL was assessed using the Italian-adapted version of the Nutrition Literacy Assessment Instrument (NLit-IT) and the adherence to the MD using MEDI-LITE. **Results**: Questionnaires filled in by 143 women were included in the analysis. Most participants (63.6%) had “possibility of poor NL”. Regarding the MD, 60.8% showed moderate and 9.1% low adherence. A positive correlation was observed between total NLit-IT and MEDI-LITE scores (rho = 0.214; *p* = 0.011). In logistic regression analysis, an increase in the NLit-IT total score was associated with a higher possibility of having a moderate/high adherence to the MD (OR 1.157). Only the subscale “Food Label and Numeracy” of NLit-IT emerged as an independent predictor of moderate/high adherence to the MD (OR 1.416). **Conclusions:** These preliminary findings suggest a possible association between NL and adherence to the MD. Further longitudinal and interventional studies are needed to confirm these results and inform tailored nutrition education interventions.

## 1. Introduction

### 1.1. Cardiovascular Diseases and Menopause

Cardiovascular diseases (CVDs) are the leading cause of mortality worldwide, accounting for an estimated 17.9 million deaths annually [1]. In Europe, CVDs are responsible for approximately 45% of all deaths among women [2], while in Italy they account for 44% of total mortality [3]. Among women, cardiovascular risk increases significantly during midlife, particularly throughout the menopausal transition. The decline in estrogen levels is linked to adverse changes in lipid profile, vascular function, and body fat distribution, contributing to an increased risk of CVDs—a fact that is often underestimated in women. Notably, around ten years after menopause, women experience a higher burden of cardiovascular events compared with men [4,5,6]. Women are particularly susceptible to both conventional and sex-specific cardiovascular risk factors, including hormonal influences and pregnancy-related conditions. In addition, socioeconomic and cultural factors may further contribute to an earlier onset and higher vulnerability to CVDs [7].

### 1.2. Mediterranean Diet and Nutrition Literacy

Among modifiable risk factors, diet plays a central role in the prevention of CVDs. The Mediterranean diet (MD) is widely regarded as one of the healthiest dietary patterns worldwide [8]. It is characterized by high consumption of whole grains, legumes, fruits, vegetables, and nuts; the use of extra virgin olive oil (EVO) as the primary source of fat; moderate consumption of white meat, fish, and dairy products; low to moderate intake of wine; and limited consumption of red and processed meats and foods rich in simple sugars [9]. In 2010, the United Nations Educational, Scientific and Cultural Organization (UNESCO) recognized the MD as an Intangible Cultural Heritage of Humanity, highlighting its cultural, social and environmental value.

A large body of evidence has demonstrated the beneficial impact of MD on multiple health outcomes, including overall mortality, CVDs, myocardial infarction, cancer, neurodegenerative diseases, diabetes mellitus, and metabolic disorders [10]. Moreover, high adherence to the MD is associated with improvements in several chronic risk factors of diseases such as total and LDL cholesterol levels, triglycerides, blood glucose, blood pressure, and waist circumference [11].

Despite these well-established benefits on health status, adherence to the MD has declined in recent decades. Many residents of Mediterranean countries now show low adherence, as globalization has shifted dietary patterns toward a Western model high in calories, saturated fats, and sugars, particularly in socioeconomically disadvantaged areas of Italy [12].

A recent report on food consumption in Europe published by the European Food Safety Authority (EFSA) [13] indicated low intake of fruit, vegetables, legumes, and milk, alongside high consumption of meat. These trends suggest that dietary behaviors may be influenced not only by food availability, but also by individual knowledge and skills.

In this context, nutrition literacy (NL) has emerged as a relevant determinant of dietary choices and health outcomes [14]. NL has been defined as a multidimensional construct and of the most adopted definitions [15] includes three main components: *Functional Nutrition Literacy* (FNL)—the ability to read and understand food labels and basic nutritional guidelines; *Interactive Nutrition Literacy* (INL)—advanced communication skills and the ability to interact effectively with nutrition professionals; *Critical Nutrition Literacy* (CNL)—the capacity to critically evaluate nutritional information, take action to improve dietary health in families and communities, and address barriers to proper nutrition.

Additionally, NL encompasses the ability to navigate the large amount of nutrition information available online [16].

Although higher levels of NL are generally associated with healthier dietary behaviors and better health outcomes, evidence regarding its relationship with adherence to the MD remains limited. In the literature, existing studies are often conducted in specific groups of different populations [17,18], which restricts the generalizability of their findings. Consequently, a gap exists in the literature concerning the role of NL in influencing adherence to the MD, which forms the exploratory basis of our study.

The aim of this study was to describe NL, adherence to the MD, and socio-demographic characteristics in women aged 45–70 years, and to explore the association between NL and adherence to the MD.

## 2. Materials and Methods

### 2.1. Study Design and Setting

This study employed a cross-sectional design and corresponded to Step 2 of Phase 1 of the Optimising Health Literacy and Access (Ophelia) process, which focuses on identifying local strengths, needs, and priorities [19,20]. Additional details are provided in the Supplementary Materials Table S1. It was conducted within the framework of the Joint Action on Cardiovascular Diseases and Diabetes (JACARDI) initiative, developed across 18 European Union (EU) Member States, implementing 142 pilot studies to improve Health Literacy (HL), self-management, screening, and primary prevention [21,22]. This study was carried out in Le Piagge, an area of Florence characterized by a higher deprivation index than the municipal average; poorer health outcomes—particularly regarding CVD—and a diverse, foreign-born population [23].

The area is supported by a network of services centered on the Casa della Comunità, an experimental community health center, the primary healthcare facility of the Local Healthcare Unit (LHU). This facility focused on providing accessible, community-based care and on enhancing integration among health professionals and services through the promotion of HL, health promotion activities and community engagement [24].

### 2.2. Study Population

The study population consisted of women aged 45–70 years at the time of recruitment who belonged to the catchment area and attended the facility. The age range of 45–70 years was selected because in this period women have an increased cardiovascular risk due to the possibility of menopausal transition and in later midlife [25]. However, menopausal status was not directly assessed, and age was used as a proxy for this life stage. Inclusion criteria were: (i) female biological sex; (ii) age between 45 and 70 years; (iii) ability to read and understand written documents in the Italian language, as the assessment tools were administered in Italian; (iv) provision of written informed consent.

### 2.3. Recruitment and Sampling

Prior to recruitment, the study was disseminated within the community through flyers and was supported by local institutions, including the local healthcare service, and community organizations (Non-Governmental Organizations—NGOs and citizens’ associations).

Recruitment took place between October 2024 and January 2025 using a non-probabilistic convenience sampling strategy combined with a snowball method, leveraging community networks.

To encourage participation, an incentive—a plant of aromatic herbs—was offered. Eligible participants who provided written informed consent were enrolled by researchers from the University of Florence. A priori sample size was estimated at 150 women, based on previous studies using the Ophelia process, and increased to 180 to account for an anticipated 20% of incomplete questionnaires.

### 2.4. Data Collection and Variables

Data collection occurred on site, and the participants were asked to self-complete paper questionnaires. When required, the researchers provided guidance to ensure accurate and complete responses, thereby standardizing administration and minimizing missing or inconsistent data.

Data collection included a specific socio-demographic questionnaire and two validated questionnaires assessing NL and adherence to the MD.

Specifically, we collected socio-demographic variables through a specific instrument, which included questions regarding the following aspects: age (year of birth); country of birth; educational level; occupational status; and financial status—investigated with the question “*With the financial resources at your disposal—from your own or family income—how do you get to the end of the month?*”, which was rated on a 4-point Likert scale from 1 = very easy to 4 = many difficulties. Moreover, health status was assessed using two items: self-perceived health status was evaluated with the question “*How is your health in general?*”, which was rated on a 5-point Likert scale from 1 = very good to 5 = very poor and chronic diseases were evaluated with the question “*Do you suffer from one or more of the chronic diseases listed below?*”. Nutritional status was collected through Body Mass Index (BMI), calculated as weight in kilograms divided by height in meters squared (kg/m^2^). BMI was classified into three categories according to the World Health Organization (WHO) criteria: underweight (BMI < 18.5 kg/m^2^), normal weight (BMI = 18.5–24.9 kg/m^2^), overweight (BMI = 25–29.9 kg/m^2^) and obese (BMI ≥ 30.0 kg/m^2^).

#### 2.4.1. Nutrition Literacy

NL was assessed using the Italian-adapted version of the Nutrition Literacy Assessment Instrument (NLit-IT) [18]. The Nutrition Literacy Assessment Instrument (NLit) is a validated questionnaire designed to measure NL, including nutrition-related knowledge and skills, in the general population. The instrument was originally developed and validated by Gibbs et al. [26], and was later adapted and validated for the Italian population by Vettori et al. [18]. The NLit-IT comprises six subscales: “Nutrition and Health”, which measures reading comprehension of dietary guidelines (10 items); “Energy Sources in Food”, which measures knowledge of macronutrient sources in food (10 items); “Household Food Measurement”, which measures the ability to identify recommended portion sizes (9 items); “Food Label and Numeracy”, which measures the ability to apply information obtained from nutrition labels (10 items); “Food Groups”, which measures the ability to classify foods according to their nutritional category (16 items); and “Consumer Skills”, which measures the ability to navigate food products in order to make healthy food choices (9 items). One point was assigned for each correct answer, with a maximum total score of 64. Both subscale and overall scores were calculated by summing the scores obtained for each item. Total scores were categorized into three levels: “Likelihood of poor NL” (score ≤ 44), “Possibility of poor NL” (score 45–57), and “Possibility of good NL” (score ≥ 58). In the validation study conducted by Vettori et al., the NLit-IT demonstrated satisfactory internal consistency, with a Cronbach’s alpha of 0.78 (95% CI, 0.69 – 0.84) [18].

#### 2.4.2. Adherence to the Mediterranean Diet

The participants reported their food consumption behavior using the MEDI-LITE questionnaire [27,28]. The instrument considers the daily or weekly intake of typical Mediterranean foods (fruit, vegetables, cereals, legumes, and fish), non-typical Mediterranean foods (meat and cured meats and dairy products), olive oil, and alcohol.

For each item, the following scores are considered:

- Typical Mediterranean foods: 2 = highest intake, 1 = intermediate, and 0 = lowest;
- Olive oil: 2 = regular use, 1 = frequent, and 0 = occasional;
- Non-typical Mediterranean foods: 2 = lowest intake, 1 = intermediate, and 0 = highest;
- Alcohol consumption: 2 = moderate (1–2 alcohol units, AU/day), 1 = low (1 AU/day), and 0 = high (>2 AU/day).

The total MEDI-LITE score is calculated by summing the individual item scores, ranging from 0 (low adherence) to 18 (high adherence). Scores are then categorized as follows:

- Low adherence: 0–6;
- Moderate adherence: 7–12;
- High adherence: 13–18.

The MEDI-LITE score was previously validated by Sofi et al., demonstrating a significant positive correlation with the validated MedDietScore (MDS) (R = 0.70, *p* < 0.0001) [28].

### 2.5. Statistical Analysis

For continuous variables, normality was checked using the Kolmogorov–Smirnov test. Data were presented as percentages for categorical variables and as mean (Standard Deviation—SD) or median (InterQuartile Range—IQR), for continuous variables, as appropriate based on their distribution. The relationship between the MEDI-LITE and NLit-IT subscale and total scale scores was assessed by both correlation and logistic regression analyses. Correlations between the MEDI-LITE and NLit-IT scores were assessed using Spearman’s rank correlation coefficient, considering both total scores and subscale/item-level scores. Because the correlation analyses were exploratory, no formal adjustment for multiple comparisons was applied. Then, logistic regression analyses were performed with the MEDI-LITE categories as the dependent variable (moderate/high vs. low adherence) and NLit-IT as the independent variable.

Two models were tested. Model 1 included the NLit-IT total score to evaluate the association between overall NL and adherence to the MD. Model 2 included the NLit-IT subscale scores significantly correlated with the MELI-LITE score, to explore whether specific domains of NL were independently associated with the outcome. This is because, for the subscale-based model, a parsimonious approach was considered to reduce model instability and collinearity and was intended to provide a more detailed understanding of which dimensions of NL might be more closely related to dietary adherence, so as to provide useful suggestions for future initiatives aimed at promoting specific components of NL. For each model, odds ratios (ORs) with 95% confidence intervals (CIs) were estimated after adjustment for the selected socio-demographic variables, namely age, country of birth, educational level, occupational status, and financial status, which were included as potential confounders because of their possible association with both NL and dietary behaviors. Only the questionnaires with complete responses to all items of the NLit-IT and MEDI-LITE were included in the analyses presented in this manuscript.

All the analyses were performed using IBM SPSS Statistics version 29.0. A *p*-value < 0.05 was considered statistically significant.

### 2.6. Ethical Consideration

The study was conducted in accordance with the Declaration of Helsinki and approved by the Ethics Committee of Tuscany Region—Central Area Vasta Centro (Comitato Etico Regionale per la Sperimentazione Clinica della Regione Toscana–Sezione AREA VASTA CENTRO) (protocol code n. 27027_oss and date of approval 3 September 2024) for studies involving humans.

## 3. Results

### 3.1. Participants’ Characteristics

Overall, 188 women were initially recruited, of whom 143 were included in the analysis. Women excluded from the analyses were significantly older than those included, whereas they did not differ in the other socio-demographic and health characteristics.

The median age of participants was 57 years (IQR: 53–63.25). As shown in Table 1, the sample was largely composed of women born in Italy (90.9%) and characterized by a medium educational level, with approximately half having completed high school (49.0%). Most participants were employed (69.2%), and more than half reported experiencing financial difficulties (52.4%). Overall, self-rated health was moderate, with the majority of women (60%) reporting fair self-perceived health status. Chronic diseases were relatively common, particularly CVDs (14.7%) and cancer (9.8%). Based on self-reported anthropometrics (2.8% missing), 35.7% were overweight (BMI 25–29.9 kg/m^2^) and 16.8% obese (BMI ≥ 30.0 kg/m^2^), indicating a high prevalence of excess body weight in the sample.

### 3.2. Nutrition Literacy

The NL levels, assessed using NLit-IT, are shown in Table 2. Overall, NL appeared to be limited in this sample. A substantial proportion of women (63.6%) were classified as having the “possibility of poor NL”, and a further 30.1% as having a “likelihood of poor NL”. Only a small proportion of women (3.5%) had the “possibility of good NL”.

At the subscale level, NLit-IT scores varied across domains. The lowest proportion of correct responses were observed for “Household Food Measurement” (58.4%) and “Food Label and Numeracy” (62.7%), followed by “Consumer Skill” (70.9%), “Energy sources in food” (74.4%) and “Food Group” (76.0%), while “Nutrition and Health” showed the highest proportion of correct responses (80.3%).

### 3.3. Adherence to the Mediterranean Diet

Based on MEDI-LITE, 60.8% of the participants (*n* = 87) showed moderate adherence to the MD, 30.1% (*n* = 43) showed high adherence, and 9.1% (*n* = 13) showed low adherence. As shown in Table 3, adherence to individual components of the MD was heterogeneous. A relatively high consumption of non-typical Mediterranean foods was observed, with 28.8% of the women reporting at least one portion/day of meat and cured meats, and 52.5% of the women reported a high intake of dairy products. In contrast, the intake of typical Mediterranean foods, such as fish, legumes, cereals, fruit and vegetables, was generally low. Specifically, 63.0% of the women consumed fewer than 2.5 portions of fish/week and 80.9% consumed no more than 1.5 portions of legumes/week. Similarly, only 26.6% of the sample reported a regular intake of cereals (>2 portions/day), while low consumption of fruit (<1 portion/day) and vegetables (<1 portion/day) was reported by 25.4% and 16.2% of the participants, respectively. Consequently, only 30.3% of the women consumed more than 2 portions/day of fruit and 40.8% more than 2.5 portions/day of vegetables. Notably, most participants (77.5%) reported using extra virgin olive oil as their main culinary fat, consistent with the Mediterranean dietary pattern.

Overall, these findings highlight an imbalanced adherence to the MD, characterized by high olive oil consumption but suboptimal intake of several core food groups.

### 3.4. Association Between Nutrition Literacy and Adherence to the Mediterranean Diet

Spearman correlations between the NLit-IT (subscales and total score) and MEDI-LITE scores are reported in Table 4. Overall, a positive correlation was observed between the NLit-IT and MEDI-LITE total scores (rho = 0.214; *p* = 0.011), indicating that higher NL was correlated with higher adherence to the MD. At the subscale level of NLit-IT, the “Food Label and Numeracy” score was the only dimension significantly correlated with MEDI-LITE total score (rho = 0.198; *p* = 0.019).

A Spearman correlation analysis was also performed at the MEDI-LITE item level as an exploratory secondary analysis, and the results should be interpreted with caution (Supplementary Materials Table S2). The consumption of fruit, vegetables, and olive oil showed statistically significant positive correlations with some subscales of NLit-IT (Supplementary Materials Table S2). Specifically, for **fruit** consumption we observed significant positive correlations with some subscales of NLit-IT, such as “Nutrition and Health” (rho = 0.202; *p* = 0.016), “Energy Sources in Food” (rho = 0.181; *p* = 0.033), “Food Label and Numeracy” (rho = 0.248; *p* = 0.003), “Food Groups” (rho = 0.279; *p* < 0.001), and the overall NLit-IT score (rho = 0.250; *p* = 0.003). **Vegetable** intake was also positively correlated with some subscales of NLit-IT, including “Nutrition and Health” (rho = 0.196; *p* = 0.020), “Food Label and Numeracy” (rho = 0.189; *p* = 0.026), “Consumer Skills” (rho = 0.191; *p* = 0.025), and the total NLit-IT score (rho = 0.231; *p* = 0.006). Finally, **olive oil** consumption showed positive correlations with the subscale “Nutrition and Health” (rho = 0.207; *p* = 0.014) and the NLit-IT total score (rho = 0.191; *p* = 0.025).

Overall, these findings suggest that higher levels of NL are significantly related to greater consumption of fruit, vegetables, and olive oil. The remaining MEDI-LITE foods (such as cereals, legumes, fish, meat and cured meats, dairy products and alcohol) showed non-significant correlations.

Table 5 reports the results of the logistic regression analyses examining the association between NL and adherence to the MD, adjusted for age, country of birth, educational level, occupational status, and financial status. In both models, the outcome variable was adherence to the MD, categorized according to the MEDI-LITE score as low versus moderate/high adherence.

In Model 1, the NLit-IT total score was significantly associated with adherence to the MD: for each one-point increase in the NLit-IT total score, the odds of having moderate/high rather than low adherence increased by approximately 16% (OR = 1.157; 95% CI: 1.052–1.273; *p* = 0.003).

In Model 2, the “Food Label and Numeracy” subscale was used instead of the total score, since it was the only subscale significantly correlated with the MEDI-LITE score in the correlation analysis. It was significantly associated with adherence to the MD (OR = 1.251; 95% CI: 1.041–1.503; *p* = 0.017).

Overall, these findings suggest that higher overall NL was associated with greater adherence to the MD. Among the specific subscales of NL, “Food Label and Numeracy” appeared to be the most relevant; however, this result should be interpreted with caution, given the exploratory nature of the subscale-based analysis and the borderline statistical significance of this association.

## 4. Discussion

This cross-sectional study represents Phase 1 (step 2) of the pilot project “Ophelia in Florence”, based on a systematic and participatory approach recommended by WHO to reduce the burden of NCDs [21,22]. The majority of participants were classified as having a “possibility of poor NL”, whereas only a limited proportion showed a “possibility of good NL”. Adherence to the MD was overall moderate. A statistically significant positive association between NL and adherence to the MD was observed.

The low levels of NL identified suggest the presence of barriers affecting both access to nutrition-related information and its effective utilization in daily dietary practices. Specifically, lower scores in the “Household Food Measurement” and “Food Label and Numeracy” subscale levels indicated deficits in functional and critical NL skills, which are essential for accurate portion size estimation, label interpretation, and informed food choices.

These findings align with previous studies reporting low to moderate levels of NL and a moderate adherence to the MD [18,29,30].

Moreover, previous studies highlighted that higher NL has been consistently associated with better food label interpretation, healthier dietary behaviors, and greater adherence to sustainable dietary patterns, including the MD [14,17]. Importantly, the present study extends the existing literature by focusing on women aged 45–70 years, a population subgroup at increased risk of CVDs. A substantial body of evidence has also highlighted the health benefits of the MD, particularly in reducing the risk and impact of mortality, CVDs, myocardial infarction, cancer, neurodegenerative diseases, diabetes mellitus, and metabolic disorders [10]. In logistic regression analysis, NL was positively correlated with adherence to the MD: higher NLit-IT scores were linked to higher MEDI-LITE scores, even after adjustment for socio-demographic characteristics.

Moreover, the observed positive association between NL and adherence to the MD supports the hypothesis that NL may influence dietary behaviors and indirectly reduce cardiovascular risk. Consistent with our findings, Yavaş et al. (2024) reported a significant positive association between NL and adherence to the MD in a sample of 3459 Turkish adults, assessed with the Evaluation Instrument of Nutrition Literacy on Adults (EINLA) and Mediterranean diet adherence screener (MEDAS) [17]. Similarly, Taylor et al. (2019) demonstrated that higher NL (measured using NLit) was associated with better overall diet quality, including a higher intake of typical Mediterranean food [14]. The Italian validation study of NLit-IT also reported that participants with higher NL scores were more likely to adhere to the MD assessed using MEDI-LITE, indicating a positive association between NL and healthy dietary behaviors [18]. However, due to the cross-sectional design of the study, causal inference and directionality of the observed associations cannot be established. Future longitudinal studies will help clarify the role of NL in shaping eating behaviors.

Our study found that, among the NLit-IT subscale levels, only “Food Label and Numeracy” was independently associated with higher adherence to the MD. Similarly, higher scores in the “Food Label and Numeracy” subscale were associated with higher adherence to the MD.

Overall, these findings suggest a possible association between NL and dietary behaviors among women aged 45–70 years, a subgroup potentially vulnerable to cardiometabolic risk.

In a socioeconomically disadvantaged area, low levels of NL were observed, particularly in the “Household Food Measurement” and “Food Label and Numeracy” subscale levels of NLit-IT, which reflect skills related to portion estimation, food label interpretation, and numeracy, were common and associated with lower adherence to the MD.

Although the cross-sectional nature of this pilot study does not allow any inference about causality or directionality, these results highlight the possible potential relevance of specific subscale levels of NLit-IT, especially food label reading and numeracy, in shaping dietary behaviors in this population. Therefore, these preliminary findings may be useful for informing future studies and for guiding the design of tailored nutrition education interventions addressing local needs. In particular, interventions aimed at improving food label reading and numeracy skills could be explored as potential strategies to promote healthier dietary patterns among women in this age group. Further longitudinal and interventional studies with larger samples are required to confirm these findings and clarify the role of NL in shaping dietary behaviors over time.

Building on these findings, the subsequent step of Phase 1 of the Ophelia process will involve participants in focus groups and participatory workshops aimed at generating ideas for action and co-design group-based nutrition education interventions, which will then be implemented in Phase 2.

This study presents several strengths and limitations. A key strength is the use of validated instruments for the Italian population (NLit-IT and MEDI-LITE), which allow for a reliable assessment of NL and adherence to the MD within the local context. Another strength is the focus on women aged 45–70 years, a population subgroup at increased cardiovascular risk due to declining estrogen levels [4]. However, several limitations should be acknowledged. First, the pilot nature of the study, the relatively small sample size, and recruitment from a single setting, which limit the generalizability of the findings. Second, the overall length of the questionnaire administration (approximately one hour), particularly due to the length of the NLit-IT, may have affected response accuracy across subscales due to potential respondent fatigue. In addition, voluntary participation may have introduced self-selection bias. The use of self-reported measures (e.g., perceived health and BMI) may have also resulted in recall and social desirability biases. Moreover, the cross-sectional design allows only identification of associations but does not permit conclusions regarding causal relationships. Finally, the logistic regression analysis of the association between NLit-IT and MEDI-LITE was based on a very small low-adherence group. Therefore, these results should be interpreted with caution, particularly in light of the borderline statistical significance observed, which may indicate potential model instability.

## 5. Conclusions

To our knowledge, this is one of the first studies to investigate the association between NL and adherence to the MD in women aged 45–70 years living in a socioeconomically disadvantaged area. By combining a multidimensional measure of NL with an assessment of adherence to the MD, this study provides preliminary evidence of a weak but statistically significant association between higher NL and greater adherence to the MD. In particular, the subscale “Food Label and Numeracy” emerged as the most consistently associated with adherence to the MD.

From a public health perspective, these results support the development of tailored, community-based nutrition education interventions, particularly in socially deprived contexts, with a focus on practical skills such as food label reading, numeracy, and portion estimation. Longitudinal and interventional studies are needed to confirm these observations and to assess whether strengthening NL can improve dietary behaviors over time.

## Figures and Tables

**Table 1:** Socio-demographic characteristics, health status and nutritional status of women included (*n* = 143).

Variables		*n*	%
**Socio-demographic characteristics**			
Country of birth	Italy	130	90.9
	Other	13	9.1
Educational level	Less than high school	32	22.4
	High school diploma	70	49.0
	Bachelor’s or master’s degree	41	28.7
Occupational status	Retired	32	22.4
	Employed	99	69.2
	Inactive	11	7.7
	Missing	1	0.7
Financial status *	Very easy or quite easy	65	45.4
	With some or many difficulties	75	52.4
	Missing	3	2.1
**Health status**			
Self-perceived health status *	Very good	9	6.3
	Good	42	29.4
	Fair	86	60.1
	Poor	6	4.2
	Very poor	0	0
	Missing	0	0
Chronic diseases	CVDs	21	14.7
	Cancer	14	9.8
	Depression and anxiety	14	9.8
	Diabetes mellitus	9	6.3
**Nutritional status**			
BMI	Underweight	6	4.2
	Healthy weight	58	40.6
	Overweight	51	35.7
	Obese	24	16.8
	Missing	4	2.8

**Legend**: Financial status investigated with * “With the financial resources at your disposal—from your own or family income—how do you get to the end of the month?”. Self-perceived health status investigated with * “How is your health in general?”. **Abbreviations**: BMI, Body Mass Index; CVDs, Cardiovascular Diseases.

**Table 2:** Distribution of descriptive statistics of NL levels, according to the NLit-IT levels of women included (*n* = 143).

NL		*n*	%
**NLit-IT**	“Likelihood of poor NL”	43	30.1
	“Possibility of poor NL”	91	63.6
	“Possibility of good NL”	5	3.5
	Missing	4	2.8

**Abbreviations**: NLit-IT, Italian-adapted version of the Nutrition Literacy Assessment Instrument. NL, Nutrition Literacy.

**Table 3:** Distribution of food consumption frequencies according to MEDI-LITE questionnaire responses among women (*n* = 143).

**Fruit**	<1 portion/day	1–2 portion/day	>2 portion/day
	25.4%	44.4%	30.3%
**Vegetables**	<1 portion/day	1–2.5 portion/day	>2.5 portion/day
	16.2%	43.0%	40.8%
**Cereals**	<1 portion/day	1–2 portion/day	>2 portion/day
	26.6%	46.8%	26.6%
**Legumes**	<1 portion/week	1–1.5 portion/week	>1.5 portion/week
	38.3%	42.6%	19.1%
**Fish**	<1 portion/week	1–2.5 portion/week	>2.5 portion/week
	32.2%	30.8%	37.1%
**Meat and meat products**	>1.5 portion/day	1–1.5 portion/day	<1 portion/day
	4.9%	23.9%	71.1%
**Dairy products**	>1.5 portion/day	1–1.5 portion/day	<1 portion/day
	11.2%	41.3%	47.6%
**Alcohol**	>2 AU/day	<1 AU/day	1–2 AU/day
	1.5%	89.0%	9.6%
**Olive oil**	Occasional	Frequent	Regular
	7.0%	15.5%	77.5%

**Legend**. Values are expressed as percentages of participants. Portion sizes refer to standard serving sizes as defined by the questionnaire. Colors indicate frequency of consumption categories: For typical Mediterranean food—red = low intake, yellow = moderate, green = high intake, according to MEDI-LITE scoring. For non-typical Mediterranean food—red = high intake, yellow = moderate, green = low intake. For alcohol: red = >2 AU/day, yellow = <1 AU/day and green = 1–2 AU/day. For olive oil—red = occasional, yellow = frequent, green = regular. **Abbreviations**: AU, alcohol unit.

**Table 4:** Spearman correlation analysis between NLit-IT and MEDI-LITE scores.

NLit-IT (Subscale and Total Score)	MEDI-LITE Total Score (rho; *p*-Value)
**Nutrition and Health**	0.130 (0.123)
**Energy Sources**	0.095 (0.267)
**Household Food Measurement**	−0.038 (0.654)
**Food Label and Numeracy**	**0.198 (0.019)**
**Food Groups**	0.146 (0.086)
**Consumer Skills**	0.114 (0.182)
**Total score**	**0.214 (0.011)**

**Abbreviations**: NLit-IT, Italian-adapted version of the Nutrition Literacy Assessment Instrument.

**Table 5:** Logistic regression analysis of the association between NLit-IT and MEDI-LITE.

Model	OR	95% CI	*p*-Value
**Model 1**			
NLit-IT score	**1.157**	**1.052–1.273**	**0.003**
**Model 2**			
Food Label and Numeracy	**1.251**	**1.041–1.503**	**0.0017**

**Legend**: Logistic regression analysis, adjusted for age, country of birth, educational level, occupational status, and financial status. Outcome: MEDI-LITE category (moderate/high vs. low). Predictors: model 1, NLit-IT total score; model 2, NLit-IT subscales. **Abbreviations**: NLit-IT, Italian-adapted version of the Nutrition Literacy Assessment Instrument; OR, odds ratio; CI, confidence interval.

## Data Availability

The data presented in this study are available upon request from the corresponding author due to privacy restrictions. The data are stored in an encrypted Excel file on a password-protected computer at the Department of Health Sciences, University of Florence. Access is restricted to authorized members of the research team.

## Supplementary Materials

The following supporting information can be downloaded at: https://www.mdpi.com/article/10.3390/nu18081238/s1, Table S1: Overview of the Ophelia process; Table S2: Spearman correlation analysis between NLit-IT and MEDI-LITE scores.

## Abbreviations

The following abbreviations are used in this manuscript:

CVD	Cardiovascular Disease
MD	Mediterranean Diet
EVO	Extra Virgin Olive Oil
UNESCO	United Nations Educational, Scientific and Cultural Organization
EFSA	European Food Safety Authority
NL	Nutrition Literacy
*FNL*	*Functional Nutrition Literacy*
*INL*	*Interactive Nutrition Literacy*
*CNL*	*Critical Nutrition Literacy*
Ophelia	Optimising Health Literacy and Access
JACARDI	Joint Action on CARdiovascular Diseases and Diabetes
EU	European Union
NCDs	Non-Communicable Diseases
HL	Health Literacy
LHU	Local Healthcare Unit
NGOs	Non-Governmental Organizations
BMI	Body Mass Index
WHO	World Health Organization
NLit-IT	Italian-adapted version of the Nutrition Literacy Assessment Instrument
NLit	Nutrition Literacy Assessment Instrument
MDS	Med Diet Score
AU	Alcohol Unit
SD	Standard Deviation
ORs	Odds Ratios
IQR	InterQuartile Range
CIs	Confidence Intervals
EINLA	Nutrition Literacy on Adults
MEDAS	Mediterranean Diet Adherence Screener
