# Supplementary material for: Nutrition Literacy and Adherence to the Mediterranean Diet in Women Aged 45–70 Years: A Cross-Sectional Analysis from the Ophelia Study in Florence
Source: Nutrients. 2026 Apr 15;18(8):1238. doi: 10.3390/nu18081238 (PMC13118363; doi:10.3390/nu18081238)
Supplement: Supplementary file 1 [file nutrients-18-01238-s001.zip › Final Supplementary Materials.pdf]

nutrients

MDPI

Nutrition Literacy and Adherence to the Mediterranean Diet in Women Aged 45–70 Years: A Cross-Sectional Analysis from the Ophelia Study in Florence

Supplementary Materials, Table S1. Overview of the Ophelia process.

Element	Description
**Overall**	The Ophelia process is a co-design methodology originally developed in Australia to support the development of HL informed interventions tailored to local contexts. The approach is aligned with strategies promoted by the WHO, which recognize HL as a key determinant in the prevention and control of NCDs.
**Main objectives**	• Reduction of health inequities associated with low HL;<br>• Creation of enabling environments that facilitate access to, understanding of, and use of health information;<br>• Promotion of sustainable changes.
**Methodological approach**	Integrated and participatory approach based on co-design, community engagement, and multisectoral coordination.
**Phase 1: "Identifying local strengths, needs and issues"**	Objectives: to collect HL and other data from community members or target populations; to discuss findings in workshops to generate ideas for HL actions; to identify effective local practices and innovative solutions. Steps:<br>1. Project set up<br>2. Data collection and extraction<br>3. Co-design workshop
**Phase 2: "Co-design of intervention"**	Objectives: to select and plan HL action (based on Phase 1 findings) to build on strengths, address needs, and improve outcomes; to test and refine selected actions.<br>Steps:<br>1. Intervention design<br>2. Intervention planning<br>3. Intervention refinement
**Phase 3: "Implementation, evaluation and ongoing improvement"**	Objectives: to implement selected HL actions; to evaluate and improve on an ongoing basis.<br>Steps:<br>1. Implementation and evaluation activities<br>2. Development of an ongoing quality improvement strategy

Abbreviations: Ophelia, Optimising health literacy and access; HL, Health Literacy; WHO, World Health Organization; NCDs, non-communicable diseases.

Supplementary Materials, Table S2. Spearman correlation analysis between NLit-IT and MEDI-LITE scores.

MEDI-LITE item / score	NLit-IT Rho (p-value)						
	Nutrition and Health	Energy Sources	Household Food Measurement	Food Label and Numeracy	Food Groups	Consumer Skills	Total score
**Fruit**	**0.202 (0.016)**	**0.181 (0.033)**	-0.022 (0.794)	**0.248 (0.003)**	**0.279 (<0.001)**	0.038 (0.661)	**0.250 (0.003)**
**Vegetables**	**0.196 (0.020)**	0.115 (0.177)	-0.065 (0.448)	**0.189 (0.026)**	0.143 (0.093)	**0.191 (0.025)**	**0.231 (0.006)**
**Cereals**	0.030 (0.724)	0.098 (0.257)	0.139 (0.106)	0.087 (0.315)	0.056 (0.522)	-0.005 (0.952)	0.104 (0.230)
**Legumes**	0.119 (0.163)	0.043 (0.615)	-0.001 (0.989)	0.130 (0.128)	0.015 (0.861)	0.141 (0.101)	0.114 (0.184)
**Fish**	-0.050 (0.555)	0.022 (0.795)	-0.058 (0.493)	0.064 (0.454)	-0.004 (0.963)	0.017 (0.843)	0.028 (0.741)
**Meat and meat products**	-0.073 (0.387)	-0.044 (0.607)	0.007 (0.935)	0.105 (0.219)	0.015 (0.864)	0.059 (0.489)	0.070 (0.417)
**Dairy products**	-0.015 (0.858)	-0.078 (0.360)	-0.082 (0.334)	-0.066 (0.440)	-0.028 (0.747)	-0.118 (0.168)	-0.084 (0.325)
**Alcohol**	-0.057 (0.512)	-0.154 (0.076)	**-0.237 (0.006)**	0.022 (0.804)	0.044 (0.617)	-0.022 (0.806)	-0.072 (0.411)
**Olive oil**	**0.207 (0.014)**	0.144 (0.090)	0.061 (0.473)	0.094 (0.273)	0.066 (0.442)	0.046 (0.589)	**0.191 (0.025)**

Abbreviations: NLit-IT, Italian-adapted version of the Nutrition Literacy Assessment Instrument.
